# Mesenchymal stem cell-originated exosomal lncRNA HAND2-AS1 impairs rheumatoid arthritis fibroblast-like synoviocyte activation through miR-143-3p/TNFAIP3/NF-κB pathway

**DOI:** 10.1186/s13018-021-02248-1

**Published:** 2021-02-06

**Authors:** Yuhua Su, Yajing Liu, Chao Ma, Chunxiao Guan, Xiufen Ma, Shan Meng

**Affiliations:** 1 Department of Rheumatology and Immunology, Affiliated Hospital of Weifang Medical University, NO.2428 Yuhe Road, Kuiwen District, Weifang, 261000 Shandong China; 2 Internal medicine, Yuncheng Hospital of traditional Chinese Medicine, Heze, 274700 Shandong China

**Keywords:** Rheumatoid arthritis, Mesenchymal stem cells, Exosomes, HAND2-AS1, miR-143-3p, TNFAIP3

## Abstract

**Background:**

Long non-coding RNA heart and neural crest derivatives expressed 2-antisense RNA 1 (HAND2-AS1) was found to be elevated in rheumatoid arthritis (RA) fibroblast-like synoviocytes (RA-FLSs). However, whether HAND2-AS1 functions as an exosomal lncRNA related to mesenchymal stem cells (MSCs) in RA progression is unknown.

**Methods:**

The expression of HAND2-AS1, microRNA (miR)-143-3p, and tumor necrosis factor alpha-inducible protein 3 (TNFAIP3) was detected using quantitative real-time polymerase chain reaction and Western blot. Cell proliferation, apoptosis, migration, and invasion were detected using cell counting kit-8, flow cytometry, and wound healing and transwell assays. The levels of tumor necrosis factor-α (TNF-α) and interleukins (IL)-6 were analyzed using enzyme-linked immunosorbent assay. The level of phosphorylated-p65 was examined by Western blot. The binding interaction between miR-143-3p and HAND2-AS1 or TNFAIP3 was confirmed by the dual-luciferase reporter and RIP assays. Exosomes were isolated by ultracentrifugation and qualified by transmission electron microscopy (TEM), nanoparticle tracking analysis (NTA), and Western blot.

**Results:**

HAND2-AS1 was lowly expressed in RA synovial tissues, and HAND2-AS1 re-expression suppressed the proliferation, motility, and inflammation and triggered the apoptosis in RA-FLSs via the inactivation of NF-κB pathway. Mechanistically, HAND2-AS1 directly sponged miR-143-3p and positively regulated TNFAIP3 expression, the target of miR-143-3p. Moreover, the effects of HAND2-AS1 on RA-FLSs were partially attenuated by miR-143-3p upregulation or TNFAIP3 knockdown. HAND2-AS1 could be packaged into hMSC-derived exosomes and absorbed by RA-FLSs, and human MSC-derived exosomal HAND2-AS1 also repressed above malignant biological behavior of RA-FLSs.

**Conclusion:**

MSC-derived exosomes participated in the intercellular transfer of HAND2-AS1 and suppressed the activation of RA-FLSs via miR-143-3p/TNFAIP3/NF-κB pathway, which provided a novel insight into the pathogenesis and treatment of RA.

## Highlights

1. HAND2-AS1 overexpression inhibited the proliferation, motility, and inflammation and induced apoptosis in RA-FLSs via NF-κB pathway.
2. HAND2-AS1 served as a sponge for miR-143-3p and positively regulated TNFAIP3 expression, which was a target of miR-143-3p.
3. The effects of HAND2-AS1 on RA-FLSs were partially attenuated by miR-143-3p upregulation or TNFAIP3 knockdown.
4. HAND2-AS1 could be wrapped by hMSC-derived exosomes and assimilated by RA-FLSs.
5. hMSC-derived exosomal HAND2-AS1 impeded RA-FLS tumor-like biologic behaviors via NF-κB pathway through miR-143-3p/TNFAIP3 axis.

## Introduction

Rheumatoid arthritis (RA) is a common systemic and chronic autoimmune disease, highlighted by hyperplasia, hypertrophy, and functional disability of joint structure, leading to incredibly high mortality and morbidity [1, 2]. Growing evidence has indicated that RA fibroblast-like synoviocytes (RA-FLSs) play key roles in the regulation of inflammatory response and joint destruction [3]. Targeting the activation of FLSs has been explored as a therapeutic strategy for RA treatment [4].

Long non-coding RNAs (lncRNAs) are RNA transcripts longer than ∼ 200 nucleotides in length, which can modulate gene expression by a wide diversity of mechanisms [5]. LncRNAs have been corroborated to have significant roles in complex human pathologies through regulating cell biological processes, such as proliferation, apoptosis, metastasis, metabolism, and inflammation [6–8], and the dysregulation of lncRNAs is associated with the physiological status and pathological progression of many diseases, including RA [9, 10]. LncRNA heart and neural crest derivatives expressed 2-antisense RNA 1 (HAND2-AS1) is a well-recognized tumor suppressor in different types of cancer [11–13]. Previous study found that the level of HAND2-AS1 was low in RA-FLSs, suggesting that abnormal HAND2-AS1 expression might be involved in synovial aggression and joint destruction in RA [14].

Recently, the use of exosomes as biological vehicles for lncRNAs transfer has attracted great research interest. Exosomes are small (30–150 nm) membranous spherical vesicles, which are actively released by almost all living cells [15]. They actually are natural information carriers and act as mediators in intercellular communications via delivering biofunctional cargoes, like proteins, lipids, DNA, and lncRNA, between cells, thereby impacting the behavior of recipient cells [16–18]. Mesenchymal stem cells (MSCs) are multipotent stem cells, which have been reported may be applied for the treatment of RA [19, 20]. MSCs produce abundant amounts of exosomes [20], and MSC-derived exosomes are more immunosuppressive in inflammatory arthritis [21]. Besides, emerging evidence has revealed that exosomes originated from MSCs play substantial roles in bone remodeling processes [22, 23]. However, whether HAND2-AS1 functions as an exosomal lncRNA related to MSCs in RA progression is unclear.

Herein, the objective of this study was to explore the effects and mechanism of HAND2-AS1 on the development of RA, evaluating the potential therapeutic ability of MSCs and related exosomes on RA treatment.

## Materials and methods

### Clinical samples

The synovial specimens of RA (RA) were collected from 28 RA patients who underwent knee joint replacement surgery or knee synovial debridement at the Affiliated Hospital of Weifang Medical University. All patients were diagnosed as RA according to the American College of Rheumatology classification. The normal synovial specimens were collected to serve as controls from 19 patients with severe joint trauma, and all enrolled subjects had no other joint abnormalities or systemic diseases. The samples were removed from discarded tissues and instantly kept in − 80 °C until used. The protocol of this study was permitted by the Ethics Committee of Affiliated Hospital of Weifang Medical University. Written informed consent was obtained from each patient.

### Cell culture

Human synovial cell line MH7A was purchased from Beijing Institute for Cancer Research Collection (Beijing, China) and cultured in the Dulbecco’s modified Eagle medium (DMEM; Invitrogen, Carlsbad, CA, USA) with 10% fetal bovine serum (FBS). Human bone marrow-derived MSCs were obtained from ATCC (PCS-500-012; Manassas, VA, USA) and grown in the α-modified Eagle’s medium (MEM) containing 10% FBS and 1% penicillin-streptomycin at 37 °C. All cultures were maintained in a humidified atmosphere of 5% CO_2_ at 37 °C. Passages 3 and 5 of MH7A and hMSCs were used for further experiments.

### Cell transfection

The pcDNA3.1 vector encoding HAND2-AS1 (HAND2-AS1) and empty vector (vector), TNFAIP3-specific siRNA (si-TNFAIP3), and negative control (si-NC) were synthesized by Genechem (Shanghai, China). The mimic of miR-143-3p (miR-143-3p) or mimic negative controls (miR-NC) were obtained from Ribobio (Guangzhou, China). The transfection was conducted with Lipofectamine™ 3000 (Invitrogen).

### Quantitative real-time polymerase chain reaction (qRT-PCR)

The isolation of whole-RNA was prepared by employing the TRIzol reagent (Invitrogen). RNA for RT-PCR was converted to complementary DNA using the PrimeScript™ II 1st Strand cDNA Synthesis Kit (Applied Biosystems, Foster City, CA, USA). Then, qRT-PCR was run with the SuperReal SYBR Green kit and ABI 7900 system (Applied Biosystems). The relative fold changes were calculated by comparing to the expression of glyceraldehyde-3-phosphate dehydrogenase (GAPDH) or U6 using the 2^−ΔΔCt^ method. The PCR primers used were listed:

HAND2-AS1: F 5′-GGAGTCACAGGCAGTCGTAGA-3′, R 5′-GAAGGCACAGATCATTCATGG-3′;

TNFAIP3: F 5′-TCCTCAGGCTTTGTATTTGAGC-3′, R 5′-TGTGTATCGGTGCATGGTTTTA-3′;

GADPH: F 5′-CCCACATGGCCTCCAAGGAGTA-3′, R 5′-GTGTACATGGCAACTGTGAGGAGG-3′;

miR-143-3p: F 5′-TGAGATGAAGCACTGTAGCTC-3′, R 5′-TGGTGTCGTGGAGTCG-3′;

U6: F 5′-AAAGCAAATCATCGGACGACC-3′, R 5′-GTACAACACATTGTTTCCTCGGA-3′.

### CCK-8 assay

Following indicated transfection or treatment of 48 h, MH7A cells (5 × 10^3^ cells/well) were placed in 96-well culture plates and then incubated with 10 μL cell counting kit-8 (CCK-8) solution for 2 h. At last, a microplate reader was applied to detect the absorbance at 450 nm in the indicated periods.

### Flow cytometry

MH7A cells (5 × 10^5^cell/mL) after indicated transfection or treatment of 48 h were resuspended in binding buffer (1×) and subsequently stained with Annexin V-fluorescein isothiocyanate (FITC) (10 μL) and propidium iodide (PI) (10 μL) (Becton Dickinson, San Jose, CA, USA). Finally, the examination of cell apoptosis was performed using the FACS Calibur flow cytometry (Becton Dickinson).

### Wound healing assay

MH7A cells with different treatments were placed in a 6-well plate (5 × 10^5^ cells/well) and scratched in the middle of the wells when cells grew to 80% confluent using a 200-μL pipette tip. Then, cells were washed and re-cultured in serum-free media. The width of scratch area was measured and photographed at 0 h and 24 h to analyze cell migration, respectively (40×).

### Transwell assay

Transwell chamber (8-mm pore size) coated with Matrigel (BD Bioscience) was employed to evaluate the invasion capacity of cells. Following different treatments, MH7A cells (1 × 10^5^) with 200 μL serum-free media were seeded into the upper chamber of transwell. Five hundred microliter media containing serum was added into the lower chambers. Following 24-h incubation, cells invaded to the lower surface were fixed with 4% paraformaldehyde and stained 0.1% with crystal violet (Sigma-Aldrich, St. Louis, MO, USA). Finally, the average numbers of invaded cells in five random fields were assessed by a microscope.

### Enzyme-linked immunosorbent assay (ELISA)

After appropriate treatment, the culture supernatants from MH7A cells culture were collected, then the concentrations of interleukin-6 (IL-6) and tumor necrosis factor-α (TNF-α) were assayed using commercial the ELISA kit (R&D Systems, Minneapolis, MN, USA) referring to the producer’s guidance.

### Western blot

Total proteins were extracted with RIPA lysis buffer and then qualified with the Bicinchoninic Acid (BCA) assay kit (Beyotime, Beijing, China). Thereafter, protein extracts (20–40 μg) were separated on a 10% sodium dodecyl sulfate polyacrylamide gel electrophoresis, transferred onto a nitrocellulose membrane, and then blocked with 5% milk for 1 h. Next, the membrane was probed with primary antibodies overnight at 4 °C, followed by the corresponding secondary antibody for 1 h at 37 °C. The blots were examined enhanced chemiluminescence solution (Beyotime). All antibodies were bought from Abcam (Cambridge, MA, USA): p65 (1:5000, ab16502), phosphorylated (p)-p65 (1: 5000, ab86299), TNFAIP3 (1:5000, ab92324), CD81 (1: 2000, ab109201), TSG101 (1:5000, ab125011), CD63 (1:2000, ab68418), GM130 (ab52649, 1:5000), GRP94 (1:1000, ab210960), and β-actin (1 : 5000, ab6276).

### Dual-luciferase reporter assay

The sequences of HAND2-AS1 and TNFAIP3 3′ UTR with miR-143-3p binding sites were cloned into pGL3 (Promega, Madison, WI, USA) to generate pGL3-HAND2-AS1 or TNFAIP3 mRNA wild-type/mutant-type luciferase reporter vector. Then, MH7A cells infected with miR-143-3p mimic or miR-NC were cultured in 12-well plates and co-transfected with 50 ng pGL3-UTR and 10 ng pRL-TK by Lipofectamine 2000 (Invitrogen). Finally, irefly and Renilla luciferase activities were measured employing the Dual-Luciferase Reporter Assay System.

### RNA immunoprecipitation (RIP) assay

MH7A cells were lysed using RIP lysis buffer. The IgG antibody or Argonaute 2 (Ago2) antibody was coupled to magnetic beads and incubated with cell lysates for 4 h at 4 °C, followed by incubation with protease K buffer to remove the protein. After that, the immunoprecipitated RNA was eluted and analyzed by qRT-PCR assay.

### Exosome (Exo) isolation and identification

Exosomes were isolated from the exosome-depleted medium of hMSCs infected with HAND2-AS1 or vector using 0.22-μm filtration and ultracentrifugation as previously described [24]. Based on the ratio of the volume of initial culture medium and the suspension (10:1), exosomes were resuspended with PBS. The morphology of exosomes was identified by the transmission electron microscopy (TEM) (JEM-1010, JEOL, Tokyo, Japan) (× 200), and the size distribution of exosomes was assessed using nanoparticle tracking analysis (NTA) (NanoSight NS300, Malvern Panalytical, Malvern, UK). Exosomes were resuspended in equal volume of RIPA lysis buffer to detect exosome-related marker (CD63, CD81, TSG101) and GRP94. Exosomes interacted with TRIzol reagent were applied to isolate RNA. For exosome co-cultures, MH7A cells (4 × 10^5^) were seeded in 6-well plates with 10% FBS exosome-depleted culture medium and then incubated with exosomes (50 μg/mL) for 48 h.

### Statistical analysis

All experiments were performed in triplicate. The data was displayed as the mean ± standard deviation (SD) and processed by GraphPad Prism 7 software. Student’s *t* test or one-way analysis of variance (ANOVA) was utilized to analyze significant differences. The *P* < 0.05 indicated statistically significant.

## Results

### HAND2-AS1 upregulation inhibits tumor-like biologic behaviors of RA-FLSs via NF-κB pathway

**Fig. 1:**
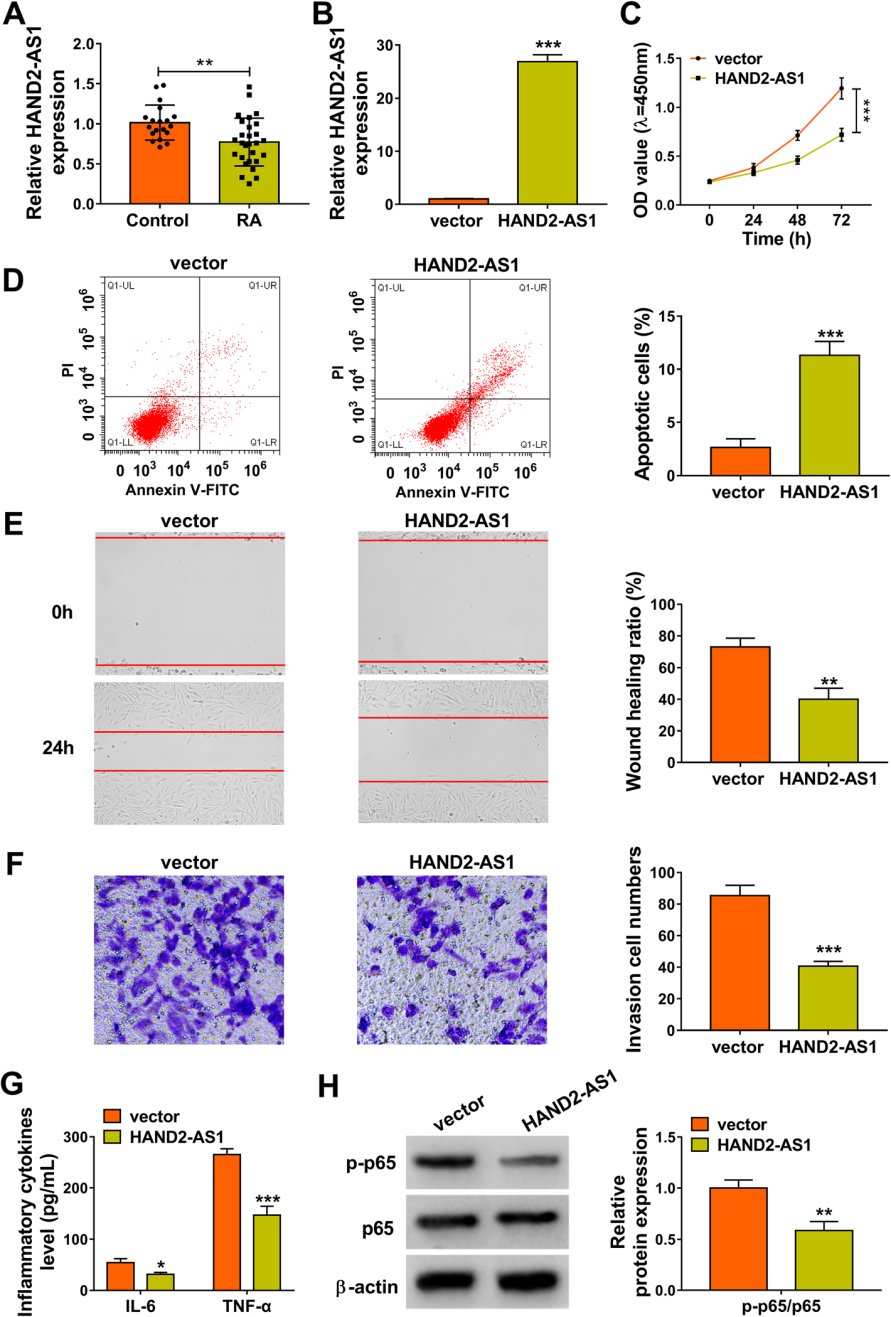
HAND2-AS1 upregulation inhibits tumor-like biologic behaviors of RA-FLSs via NF-κB pathway. **a** qRT-PCR analysis of HAND2-AS1 expression in RA synovial tissues and normal synovial tissues. **b**–**h** MH7A cells were transfected with HAND2-AS1 or vector. **b** qRT-PCR analysis of HAND2-AS1 expression in cells. **c** CCK-8 assay of cell proliferation. **d** Flow cytometric analysis of cell apoptosis. **e** Wound healing assay of cell migration. **f** Transwell assay of cell invasion. **g** Detection of IL-6 and TNF-α levels in cells with ELISA. **h** Western blot analysis of the level of p-p65 and p65. *P < 0.05, **P < 0.001, ***P < 0.0001

Initially, the expression profile of HAND2-AS1 was investigated in synovial tissues. In comparison with normal synovial specimens, HAND2-AS1 was lowly expressed in synovial tissues with RA (Fig. 1a). Next, the role of HAND2-AS1 in RA progression was studied, we transfected HAND2-AS1 plasmid into MH7A cells. qRT-PCR analysis indicated that HAND2-AS1 level in MH7A cells was significantly upregulated after cells were transfected with HAND2-AS1 (Fig. 1b). After that, a CCK-8 assay suggested that the proliferation of MH7A cells was markedly reduced by HAND2-AS1 upregulation (Fig. 1c). On the contrary, HAND2-AS1 overexpression significantly promoted apoptosis in MH7A cells (Fig. 1d). Moreover, wound healing and transwell assays were performed; we found that the migration and invasion abilities of HAND2-AS1-increased MH7A cells were markedly suppressed (Fig. 1e, f). Results of ELISA indicated HAND2-AS1 upregulation repressed inflammation of MH7A cells, reflected by the decrease of IL-6 and TNF-α levels (Fig. 1g). Besides that, the level of phosphorylated p65 (p-p65) was found to be reduced by HAND2-AS1 overexpression in MH7A cells, suggesting that HAND2-AS1 could inactivate NF-κB pathway (Fig. 1h). Taken together, HAND2-AS1 upregulation inhibited the proliferation, motility, and inflammation and induced apoptosis in RA-FLSs via the inactivation of NF-κB pathway.

### MiR-143-3p is a target of HAND2-AS1

**Fig. 2:**
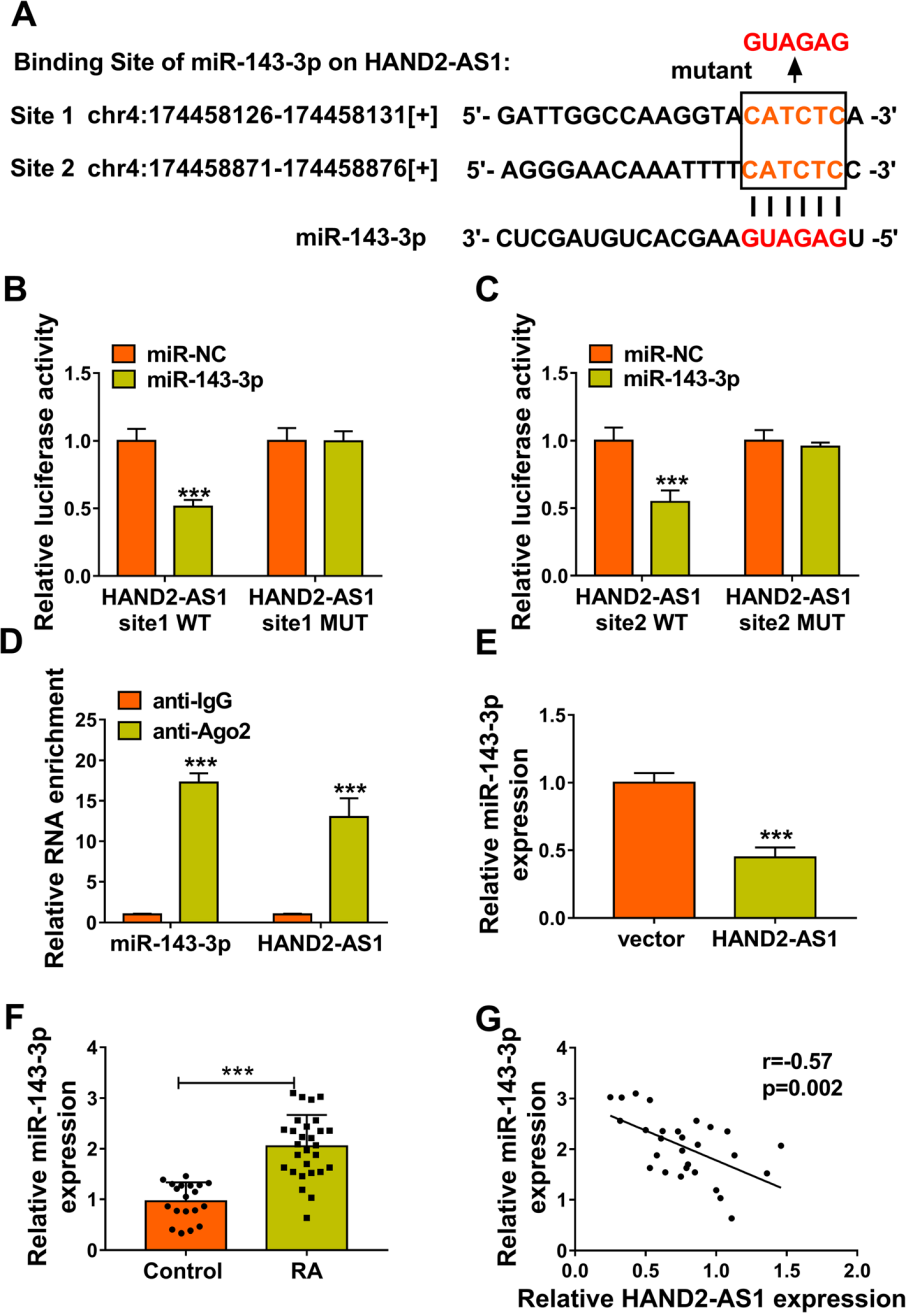
MiR-143-3p is a target of HAND2-AS1. **a** The potential binding sites of miR-143-3p on HAND2-AS1. **b**–**d** The interaction between miR-143-3p and HAND2-AS1 was confirmed using dual-luciferase reporter assay and RIP assay. **e** qRT-PCR analysis of miR-143-3p expression in MH7A cells transfected with vector or HAND2-AS1. **f** qRT-PCR analysis of miR-143-3p expression in RA synovial tissues and normal synovial tissues. **g** Pearson correlation analysis of miR-143-3p and HAND2-AS1 expression in 28 RA tissues. ***P < 0.0001

The regulatory mechanism of HAND2-AS1 in RA-FLS tumor-like biologic behaviors was then investigated. Using MiRcode bioinformatic analysis software, it was found that HAND2-AS1 possessed conserved target sites of miR-143-3p (Fig. 2a). Then, the dual-luciferase reporter assay was conducted; results showed that miR-143-3p mimics markedly reduced the luciferase activities of the wild-type HAND2-AS1 reporter, but not the mutant-type HAND2-AS1 vector in MH7A cells (Fig. 2b, c). Moreover, RIP assay confirmed that HAND2-AS1 and miR-143-3p were efficiently pulled down by anti-Ago2 antibodies compared to control IgG in MH7A cells (Fig. 2d). All these data conformed that HAND2-AS1 directly targeted miR-143-3p. In addition, we observed that miR-143-3p expression was reduced by HAND2-AS1 overexpression in MH7A cells (Fig. 2e). The expression of miR-143-3p was higher in RA synovial tissues than that in normal synovial tissues (Fig. 2f), and a negative correlation between miR-143-3p and HAND2-AS1 expression in RA synovial tissues was verified (Fig. 2g). Collectively, HAND2-AS1 targetedly suppressed miR-143-3p expression in RA-FLSs.

### MiR-143-3p reverses the effects of HAND2-AS1 overexpression on RA-FLSs

**Fig. 3:**
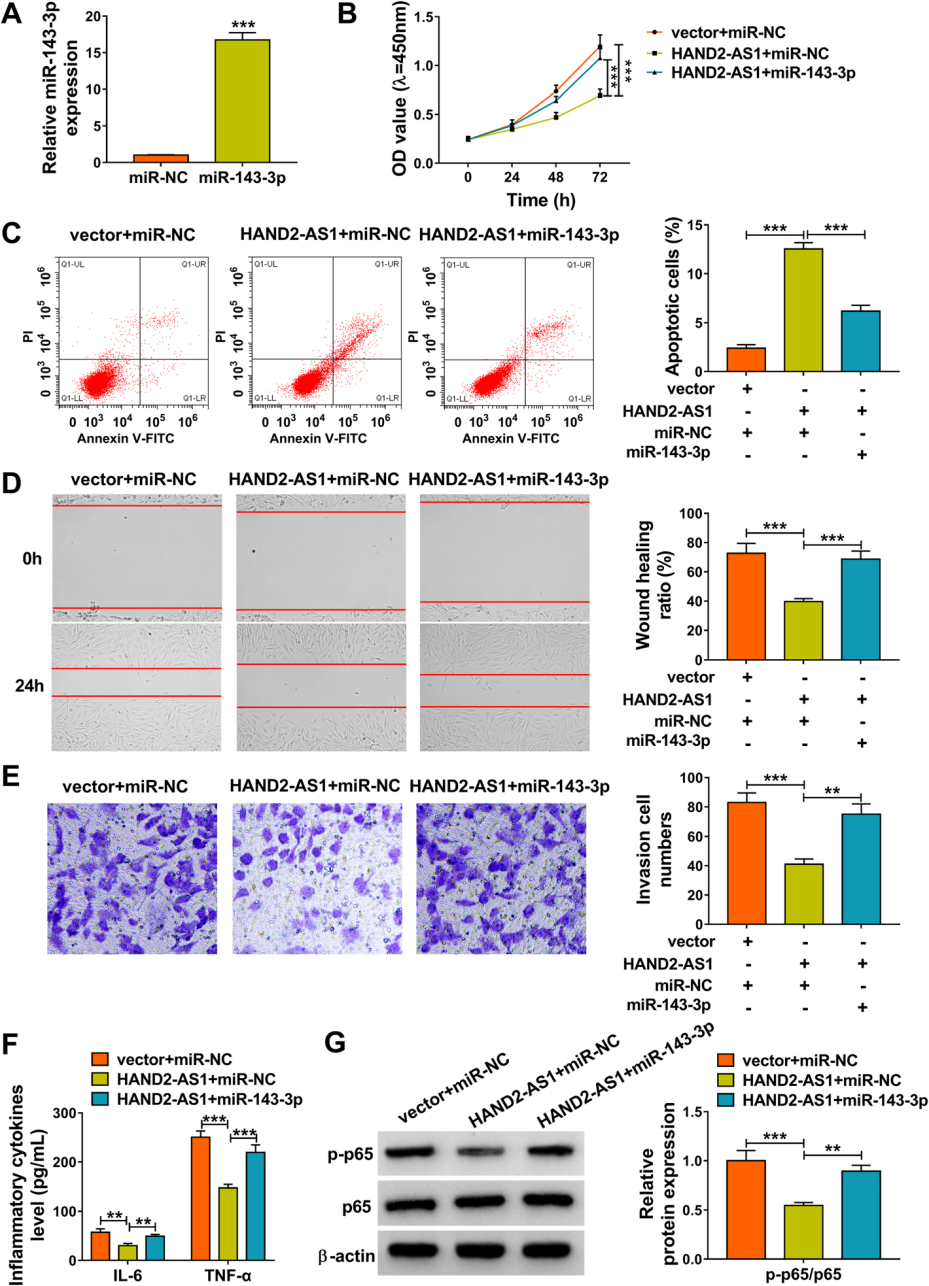
MiR-143-3p reverses the effects of HAND2-AS1 overexpression on RA-FLSs. **a** qRT-PCR analysis of miR-143-3p expression in MH7A cells transfected with miR-NC or miR-143-3p. **b**–**g** MH7A cells were transfected with vector + miR-NC, HAND2-AS1 + miR-NC, or HAND2-AS1 + miR-143-3p. **b** CCK-8 assay for cell proliferation. **c** Flow cytometry for cell apoptosis. **d** Wound healing assay for cell migration. **e** Transwell assay for cell invasion. **f** ELISA for the detection of IL-6 and TNF-α levels in cells. **g** Measurement of p-p65 and p65 levels in cell using Western blot. **P < 0.001, ***P < 0.0001

To investigate whether the effects of HAND2-AS1 on RA-FLSs were mediated by miR-143-3p, the transfection efficiency of miR-143-3p mimic was firstly investigated. As expected, miR-143-3p expression level was significantly increased by miR-143-3p mimic in MH7A cells (Fig. 3a). Then, the miR-143-3p mimic and HAND2-AS1 plasmid were co-transfected into MH7A cells. It was proved that co-transfection of miR-143-3p reversed the effects of HAND2-AS1 overexpression on the suppression of RA-FLS proliferation (Fig. 3b), migration (Fig. 3d), invasion (Fig. 3e), and inflammation (Fig. 3f), as well as the enhancement of cell apoptosis (Fig. 3c). Also, miR-143-3p upregulation increased the level of p-p65 of in HAND2-AS1-incrased MH7A cells (Fig. 3g). Therefore, we confirmed that HAND2-AS1/miR-143-3p axis was engaged in the tumor-like biologic behaviors of RA-FLSs via NF-κB pathway.

### TNFAIP3 is a target of miR-143-3p and could be indirectly regulated by HAND2-AS1

**Fig. 4:**
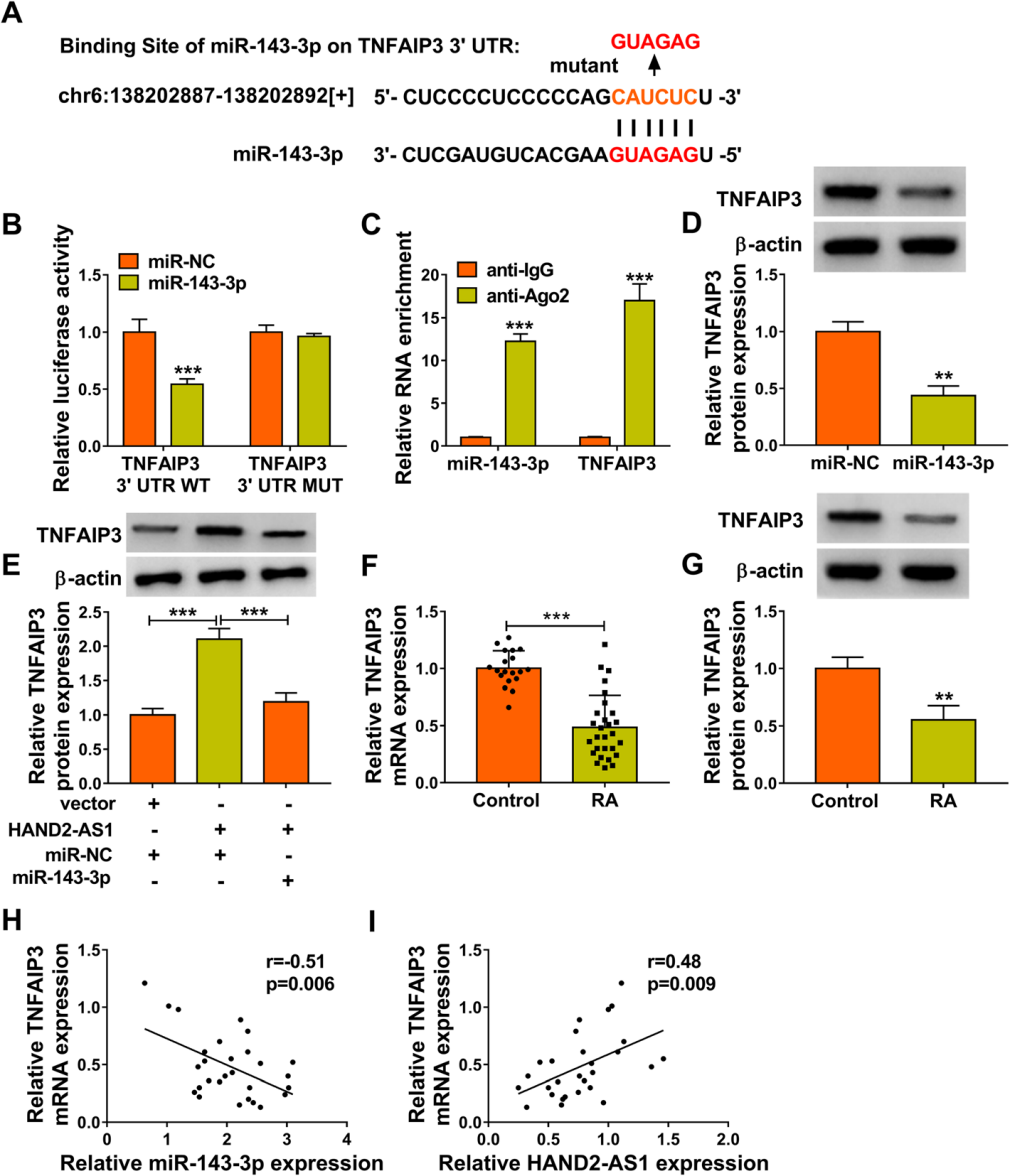
TNFAIP3 is a target of miR-143-3p and could be indirectly regulated by HAND2-AS1. **a** The potential binding sites of miR-143-3p on TNFAIP3 3′ UTR. **b**, **c** The interaction between miR-143-3p and TNFAIP3 was verified using dual-luciferase reporter and RIP assays. **d** Western blot analysis of TNFAIP3 expression in MH7A cells transfected with miR-143-3p or miR-NC. **e** Western blot analysis of TNFAIP3 expression in MH7A cells transfected with vector + miR-NC, HAND2-AS1 + miR-NC, or HAND2-AS1 + miR-143-3p. **f**, **g** qRT-PCR and Western blot analysis of TNFAIP3 expression in RA synovial tissues and normal synovial tissues. **h**, **i** Pearson correlation analysis of TNFAIP3 and miR-143-3p or HAND2-AS1 expression in 28 RA tissues. **P < 0.001, ***P < 0.0001

Through searching the starBase database, it was found that there was a potential base-complementary binding site between miR-143-3p and the TNFAIP3 3’UTR (Fig. 4a). The dual-luciferase reporter assay showed a remarkable reduction in the luciferase activity following co-transfection of MH7A cells with miR-143-3p mimics and wild-type TNFAIP3 3’UTR vector, but not with the mutant-type TNFAIP3 3′ UTR vector (Fig. 4b). RIP assay further demonstrated that TNFAIP3 and miR-143-3p were enriched by anti-Ago2 compared to control IgG (Fig. 4c). Furthermore, miR-143-3p overexpression repressed the expression of TNFAIP3 in MH7A cells (Fig. 4d). Therefore, we confirmed that miR-143-3p directly targeted TNFAIP3 and negatively regulate its expression.

In addition, we also found HAND2-AS1 overexpression in MH7A cells upregulated the level of TNFAIP3, which was abolished by miR-143-3p increase (Fig. 4e). TNFAIP3 expression was decreased in RA synovial tissues (Fig. 4f, g), which was negatively correlated with miR-143-3p expression (Fig. 4h), while positively correlated with HAND2-AS1 expression (Fig. 4i). In summary, HAND2-AS1 served as a sponge for miR-143-3p to regulate TNFAIP3 expression.

### Knockdown of TNFAIP3 reverses the effects of HAND2-AS1 overexpression on RA-FLSs

**Fig. 5:**
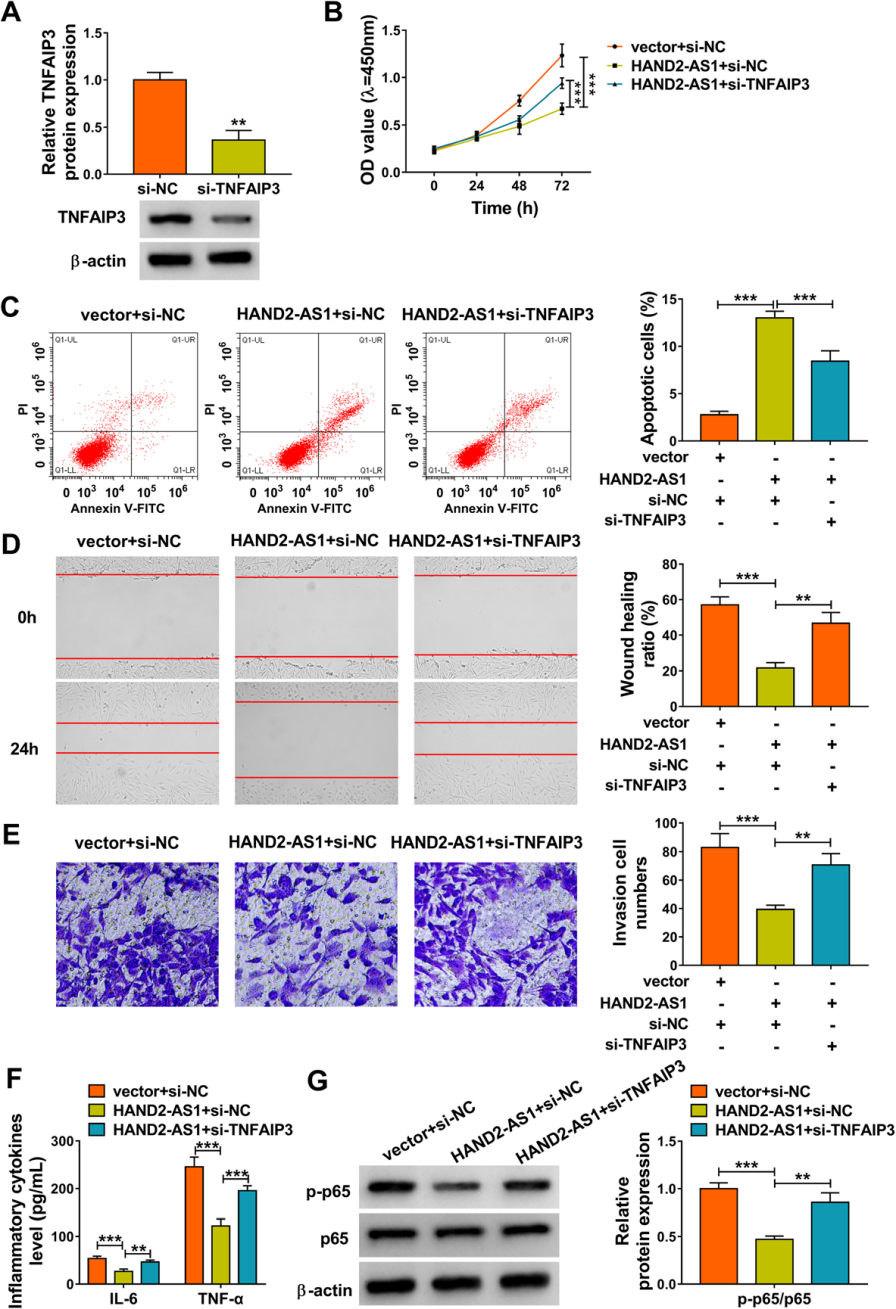
Knockdown of TNFAIP3 reverses the effects of HAND2-AS1 overexpression on RA-FLSs. **a** Western blot analysis of TNFAIP3 expression in MH7A cells transfected with si-NC or si-TNFAIP3. **b**–**g** MH7A cells were transfected with vector + si-NC, HAND2-AS1 + si-NC, or HAND2-AS1 + si-TNFAIP3. **b** CCK-8 assay of cell proliferation. **c** Flow cytometric analysis of cell apoptosis. **d** Wound healing assay of cell migration. **e** Transwell assay of cell invasion. **f** Detection of IL-6 and TNF-α levels in cells with ELISA. **g** Western blot analysis of the level of p-p65 and p65. **P < 0.001, ***P < 0.0001

Based on the aforementioned results, we further investigated whether TNFAIP3 might have roles in the action of HAND2-AS1 in RA-FLS tumor-like biologic behaviors. Firstly, we confirmed that transfection of si-TNFAIP3 markedly reduced the level of TNFAIP3 in MH7A cells (Fig. 5a). Next, si-TNFAIP3 was transfected into HAND2-AS1-overexpressed MH7A cells. Results demonstrated that TNFAIP3 knockdown contributed to the proliferation (Fig. 5b), migration (Fig. 5d), invasion (Fig. 5e), and inflammation (Fig. 5f) and suppressed the apoptosis (Fig. 5c) in HAND2-AS1-overexpressed MH7A cells. Besides that, downregulation of TNFAIP3 increased HAND2-AS1-evoked decrease of p-p65 (Fig. 5g). Altogether, HAND2-AS1 inhibited the proliferation, motility, and inflammation of RA-FLSs via the inactivation of NF-κB pathway through TNFAIP3.

### HAND2-AS1 is highly enriched in exosomes derived from HAND2-AS1-transfected hMSCs

**Fig. 6:**
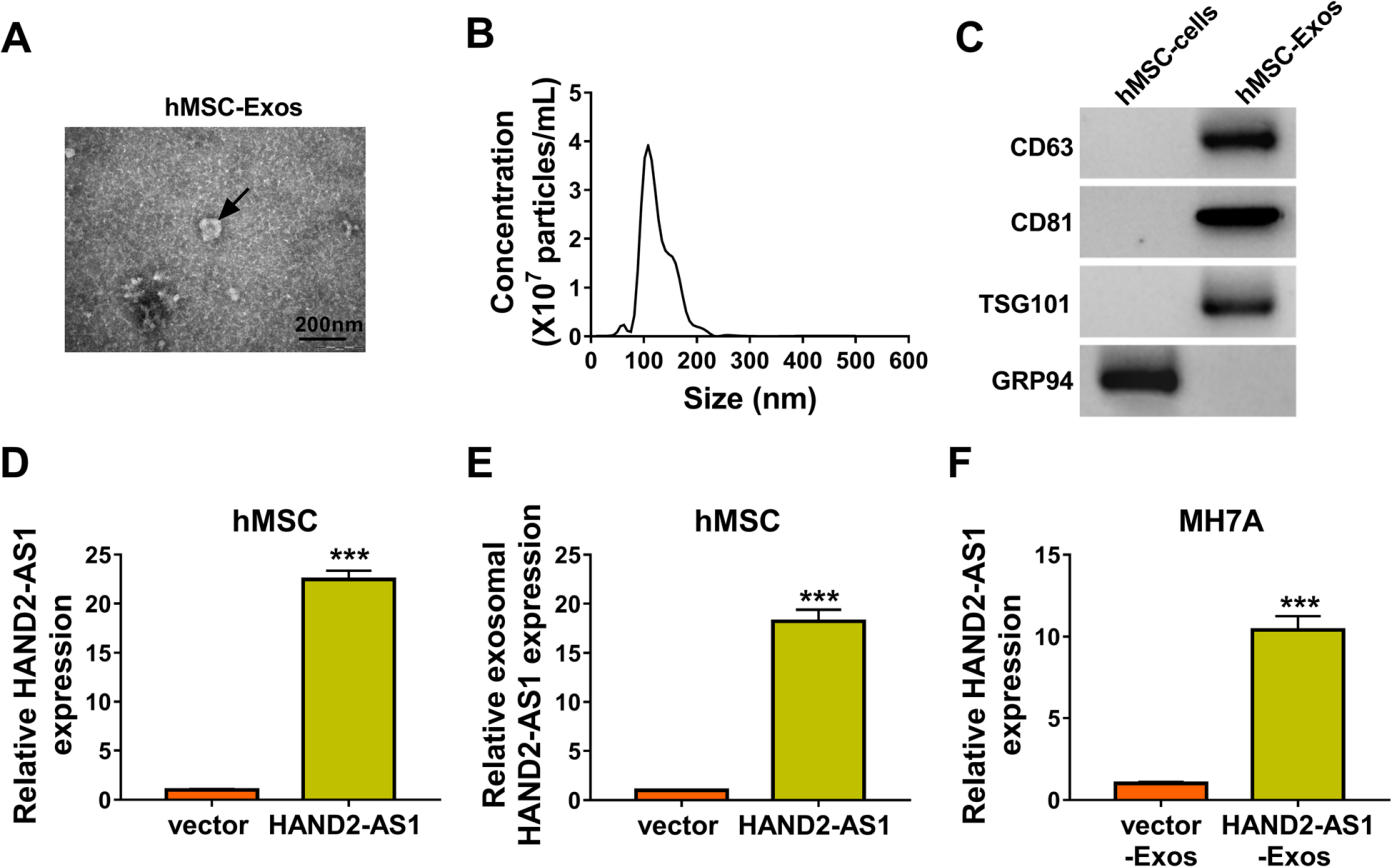
HAND2-AS1 is highly enriched in exosomes derived from HAND2-AS1-transfected hMSCs. **a** Representative TEM images of exosomes from hMSCs. **b** Exosomes isolated from hMSCs underwent a NTA to determine exosomal size distribution. **c** Western blot analysis of CD63, CD81, TSG101, and GRP94 expression in isolated exosomes and the lysate of hMSCs. **d** qRT-PCR analysis of HAND2-AS1 level in hMSCs transfected with vector or HAND2-AS1. **e** qRT-PCR analysis of HAND2-AS1expresison in exosomes derived from transfected hMSCs. **f** qRT-PCR analysis of HAND2-AS1expresison in MH7A cells co-cultured with HAND2-AS1-Exos or vector-Exos. ***P < 0.0001

In order to elucidate the effects of hMSC-derived exosomal HAND2-AS1 on RA-FLSs, the exosomes were firstly extracted from hMSCs and identified. TEM analysis confirmed the presence of translucent, cup-shaped vesicles (Fig. 6a), and nanosight tracking analysis (NAT) revealed that the main particle size of vesicles was in the 120-nm range, consistent with the unique characteristics of exosomes (Fig. 6b). Moreover, the Western blot analysis showed that hMSC-derived exosomes expressed the marker proteins CD63, CD81, and TSG101 and did not express GRP94 (Fig. 6c). qRT-PCR analysis suggested that HAND2-AS1 level was increased in hMSCs after HAND2-AS1 plasmid transfection (Fig. 6d). Then, the exosomes were extracted from hMSCs treated with HAND2-AS1; also, the level of HAND2-AS1 in exosomes derived from transfected hMSCs (HAND2-AS1-Exos) was upregulated (Fig. 6e). Next, MH7A cells were co-cultured with HAND2-AS1-Exos, and as expected, the expression of HAND2-AS1 was significantly elevated in MH7A cells (Fig. 6f). To sum up, HAND2-AS1 could be wrapped by hMSC-derived exosomes and assimilated by RA-FLSs.

### hMSC-derived exosomal HAND2-AS1 suppresses tumor-like biologic behaviors of RA-FLSs via NF-κB pathway

**Fig. 7:**
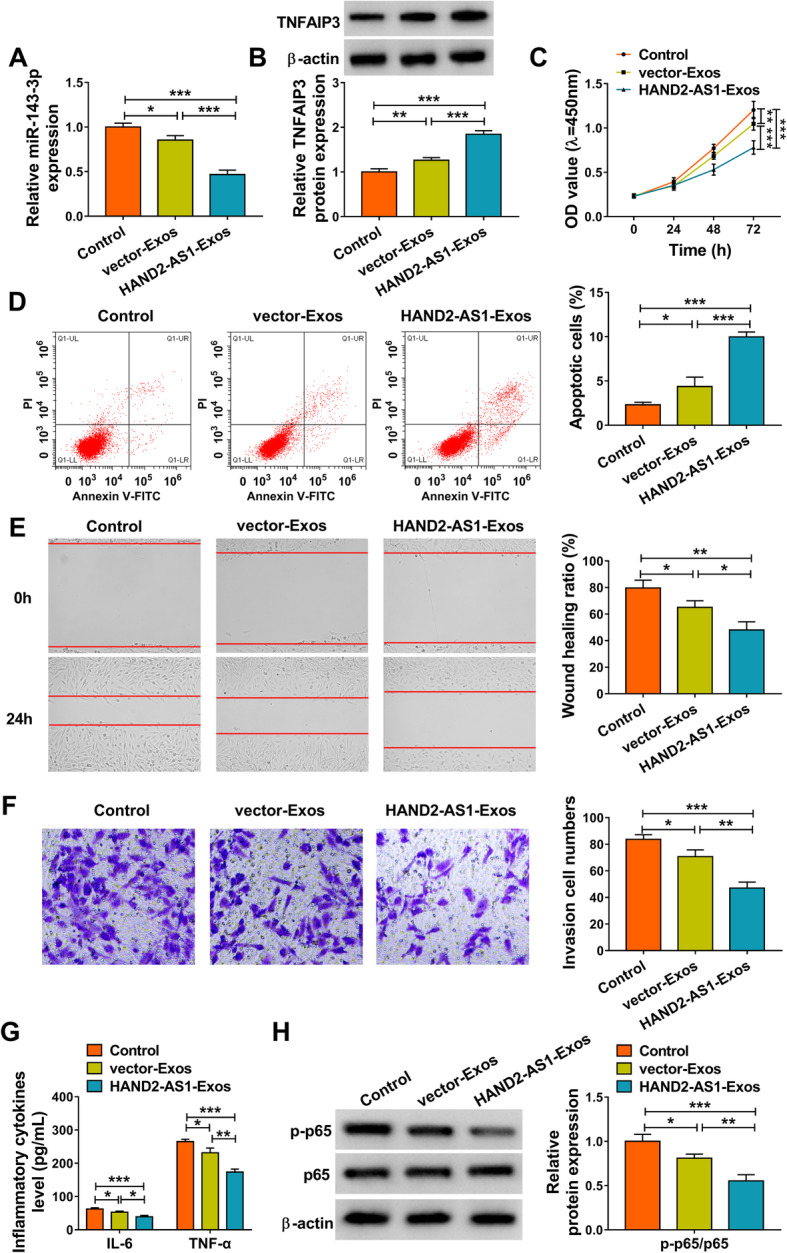
hMSC-derived exosomal HAND2-AS1 suppresses tumor-like biologic behaviors of RA-FLSs via NF-κB pathway. **a**–**h** MH7A cells were incubated with HAND2-AS1-Exos or vector-Exos. **a** qRT-PCR analysis of miR-143-3p expression in MH7A cells. **b** Western blot analysis of TNFAIP3 expression in MH7A cells. **c** Cell proliferation analysis with CCK-8. **d** Flow cytometric analysis of cell apoptosis. **e** Cell migration analysis using wound healing assay. **f** Transwell assay of cell invasion. **g** Detection of IL-6 and TNF-α levels in cells with ELISA. **h** Western blot analysis of the level of p-p65 and p65. *P < 0.05, **P < 0.001, ***P < 0.0001

**Fig. 8:**
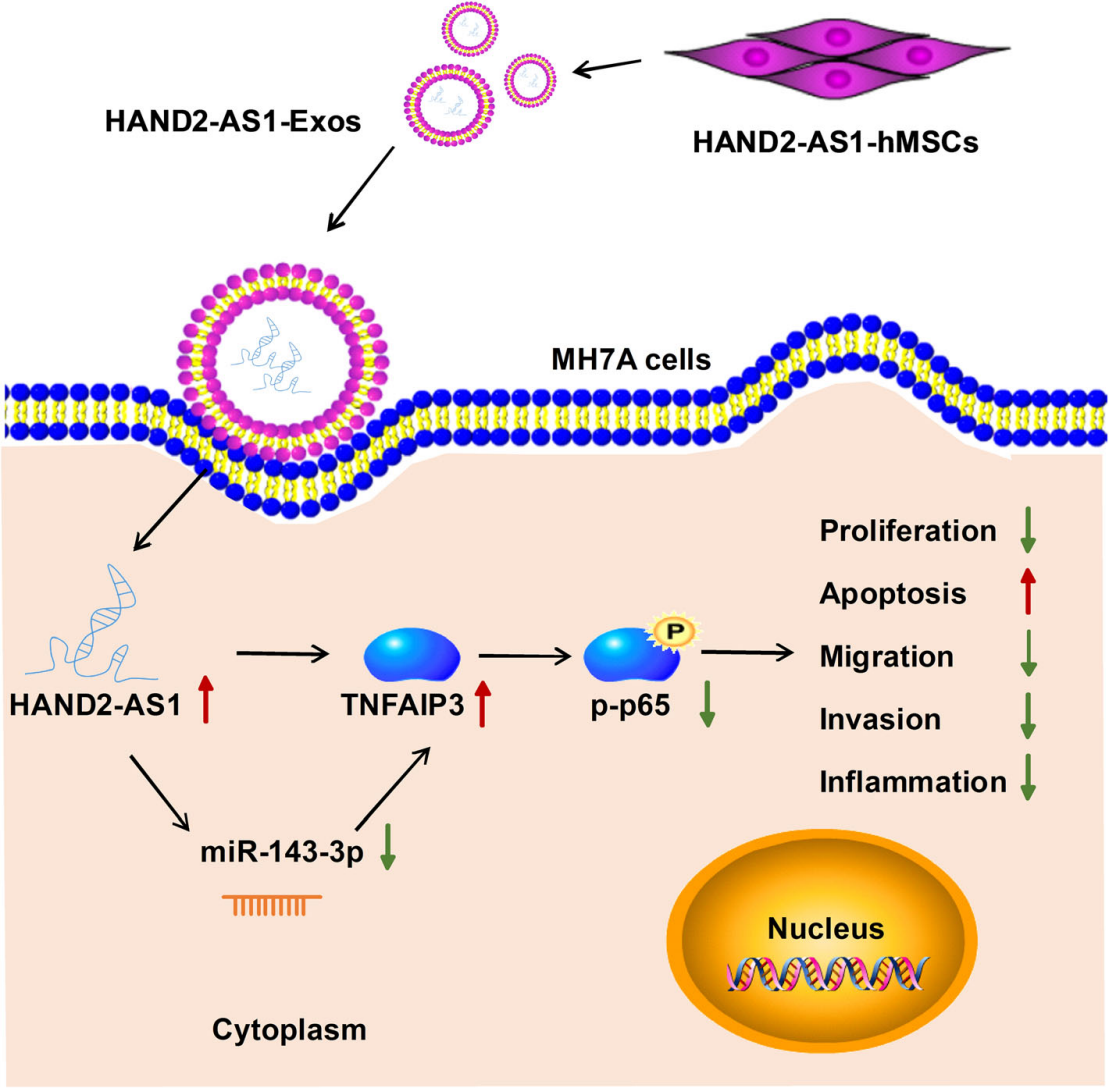
Schematic diagram of how hMSC-originated exosomal HAND2-AS1 suppresses the activation of RA-FLSs. hMSC-derived exosome-mediated transfer of HAND2-AS1 impeded RA-FLS tumor-like biologic behaviors via NF-κB pathway through miR-143-3p/TNFAIP3 axis

Subsequently, the function of exosomal HAND2-AS1 from hMSCs was investigated. After the co-culture of HAND2-AS1-Exos and MH7A cells, we also observed that the expression of miR-143-3p was apparently decreased (Fig. 7a) and TNFAIP3 was increased (Fig. 7b) in MH7A cells. Further functional studies exhibited that HAND2-AS1-Exos co-culture impaired the proliferation (Fig. 7c), migration (Fig. 7e), invasion (Fig. 7f), and inflammation (Fig. 7g) and boosted the apoptosis (Fig. 7d) in MH7A cells. Besides that, the expression of p-p65 was obviously downregulated by co-culture of HAND2-AS1-Exos in MH7A cells (Fig. 7h). These results suggested that hMSC-derived exosome-mediated transfer of HAND2-AS1 impeded RA-FLS tumor-like biologic behaviors via NF-κB pathway through miR-143-3p/TNFAIP3 axis (Fig. 8).

## Discussion

FLSs are the major cell types that make up the synovial intima structure, and RA-FLSs exhibit “tumor-like” properties, including aggressive proliferation, apoptosis arrest, and increased invasiveness, which are critical in the formation of synovial pannus; besides that, activated RA-FLSs release abundant inflammatory cytokines, chemokines, and metalloproteinases, mediating inflammation and further exacerbating joint damage, and ultimately exacerbating the progression of RA [3, 25, 26]. Therefore, an investigation of the aggressive phenotype of RA-FLSs is required to develop novel therapy regimens for RA patients.

In the current study, a low expression of HAND2-AS1 was observed in synovial tissues with RA. Then, we upregulated the level of HAND2-AS1 in RA-FLSs in vitro, and it was proved that HAND2-AS1 re-expression impaired the proliferation, invasion, migration, and inflammation and contributed to the apoptosis in RA-FLSs; thus, we knew that HAND2-AS1 was of great importance in the pathogenesis of RA. NF-κB is a ubiquitously expressed pleiotropic transcription factor present in almost all cell types; aberrant regulation of NF-κB and its downstream targets often result in inflammation, cell growth, drug resistance, and cancer metastasis [27]. p65, as one of the five components that form the NF-κB transcription factor family, is typically involved in the body’s inflammatory response [28]. In this study, we also observed that HAND2-AS1 reduced the level of p-p65 in RA-FLSs. Therefore, we concluded that HAND2-AS1 might repress RA progression via the inactivation of NF-κB pathway. MSCs possess the ability to differentiate into multiple cell lines, including osteoblasts, chondrocytes, and adipocytes; form bone and cartilage; and exert immunosuppressive functions [29–31]; besides owing to their capacity to abolish the exacerbated pathogenic immune response observed in these patients, MSCs have been considered as interesting therapeutic cell candidates for the treatment of RA [32]. Exosomes are small spherical packages, which possess the physical properties of artificial nanoparticles and additional advantages such as excellent biocompatibility, biodegradability, and sequence programmability, and are ideal drug delivery carriers [33]. In the current study, we generated hMSC-HAND2-AS1-Exos (exosomes derived from HAND2-AS1-overexpressing hMSCs); after the co-incubation of RA-FLSs with hMSC-HAND2-AS1-Exos, HAND2-AS1 level was remarkably elevated in RA-FLSs and also suppressed cell growth, mobility, and inflammation and induced cell apoptosis. Taken together, MSC-derived exosomes are involved in the intercellular transfer of HAND2-AS1 and subsequently impeded RA progression.

Previous studies have shown that HAND2-AS1 can perform a suppressive role in tumor progression by serving as a sponge of miRNAs [11, 12]. Emerging evidence has revealed that the novel regulatory network of lncRNAs-miRNAs-mRNAs plays a significant role in the pathophysiological processes of many diseases, including RA [34, 35]. Therefore, the HAND2-AS1-miRNAs-mRNAs network in RA was investigated in this study. It was confirmed that HAND2-AS1 served as a sponge for miR-143-3p and positively regulated TNFAIP3 expression, which was verified to be a direct target of miR-143-3p. Subsequent analysis suggested that the effects of HAND2-AS1 on RA-FLSs and the inactivation of NF-κB pathway were abolished by miR-143-3p overexpression or TNFAIP3 downregulation. Moreover, when the level of HAND2-AS1 was elevated in RA-FLSs through the co-culture with hMSC-HAND2-AS1-Exos, we found miR-143-3p expression was inhibited and HAND2-AS1 expression was increased; importantly, hMSC-derived exosomal HAND2-AS1 also suppressed tumor-like biologic behaviors of RA-FLSs via NF-κB pathway.

In conclusion, this study demonstrated that overexpression of HAND2-AS1 in exosomes derived from MSCs suppressed the proliferation, invasion, migration, and inflammation and induced apoptosis in RA-FLSs through the inactivation of NF-κB pathway via miR-143-3p/TNFAIP3 axis. MSCs are easily isolated and amenable to culture expansion in vitro, MSC transplantation is undergoing extensive evaluation as a cellular therapy in human clinical trials [36]; however, rejection by the host and tumorigenicity have limited its clinical application [37]. Exosomes are one of extracellular vesicles that stably present in body fluids due to their phospholipid bilayer; besides that, owing to their RNA transport capacity and ability crossing the blood brain barrier, exosomes are considered candidate drug delivery vehicles [38]. MSCs produce abundant amounts of exosomes, and MSC-derived exosomes have a content that includes cytokines and growth factors, signaling lipids, mRNAs, and regulatory miRNAs [36]. Therefore, loading specific lncRNA into MSC-derived exosomes may provide a new therapeutic paradigm for cell-free MSC-based therapies in clinic. Our study suggests a potential therapeutic strategy for the treatment of RA.

## Data Availability

All data generated or analyzed during this study are included in this published article.
